# Stomatitis Materni

**Published:** 1853-08

**Authors:** 


*Stomatitis Materni.*—" Has this disease ever existed when the symptoms of *general* disorder were not accompanied by it, traceable to either retention or suppression of bile ?" In the last 15 years I have not found it necessary to take the child from the breast, and my practice has been based on a negative answer to this question. I have witnessed two post obit. examinations of patients, who labored, at time of death, under this disease, in each the gall bladder and duct were clogged with calculi and thickend bile. In the living patients I have observed, in almost every instance, 1st, an anemic condition ; 2nd, acidity of stomach, 3d, as a consequence of the last, urinary deposits, *invariably relieved by the free use of alkalies* ; 4th, the alvine evacuations either hard or black, denoting the absence of the natural and accustomed stimulus to the bowels, or clay, colored with a tendency to diarrhœa, showing both a want of biliary presence, and the existence of fermentations, which never (I think) occurs when the proper proportion of bile is present

The presence of anæmia I have not found necessary to the existence of the disease, but if present, it greatly adds to the chances of continuance. Indeed, it often produces the very state of things, on which, I think, the disease depends. In an anæmic condition it is difficult for the system to perform all of its *usual* functions, and at the same time support the new one now set up—the support of the infant, either in embryo or by lactation. For this support a *new* draft is made upon the energies of the system, and this draft is often made upon some *one* organ, instead of beeing distributed equally on the whole; that one must, of course, lose much of its energy; and, should it be the liver, we have want of its secretion; as a consequence, we have fermentation and acidity of stomach and bowels with its sequents, amongst which is ulceration of certain of the mucous membranes; and, to the existence of this condition of the membranes, neither pregnancy nor lactation are *necessary*, as it will be found under many circumstances in the absence of both these, though *never* under my observation without the previous withdrawal of the accustomed supply of bile. The only difference to be found in the disease under these different circumstances is, that during lactation or pregnancy it is more obstinate in consequence of the draft upon the energies of the system being continuous, and consequently renewing the disease as often as the remedies for its relief are withheld, or until the vital engeries are increased by tonics, or otherwise sufficiently to meet the new demand without the special draft.—*Ibid.*

